# P16INK4A Immunohistochemistry as a Gold Standard for Cervical Cancer and Precursor Lesions Screening

**Published:** 2020-02

**Authors:** Mahdieh FARZANEHPOUR, Ahad MUHAMMADNEJAD, Setareh AKHAVAN, Amir Nader EMAMI RAZAVI, Somayeh JALILVAND, Vahid SALIMI, Ebrahim FAGHIHLOO, Ehsan KAKAVANDI, Mohammad FARAHMAND, Mohammad SHAYESTEHPOUR, Farzad BABAKHANI, Talat MOKHTARI AZAD

**Affiliations:** 1. Department of Virology, School of Public Health, Tehran University of Medical Sciences, Tehran, Iran; 2. Department of Microbiology, Applied Microbiology Research Center, Baqiyatallah University of Medical Sciences, Tehran, Iran; 3. Cancer Biology Research Center, Cancer Institute of Iran, Tehran University of Medical Sciences, Tehran, Iran; 4. Department of Gynecology/Oncology, Imam Khomeini Hospital Complex, Valiasr Hospital, Tehran University of Medical Sciences, Tehran, Iran; 5. Department of Microbiology, School of Medicine, Shahid Beheshti University of Medical Sciences, Tehran, Iran

**Keywords:** Human papillomavirus, p16INK4A, Immunohistochemistry

## Abstract

**Background:**

High-risk (HR) Human papillomaviruses (HPVs) are known as the main factors implicated in the pathogenesis of cervical preinvasive and invasive lesions. Therefore, the presence or absence of HR-HPV can be followed for the prognosis of low-grade and high-grade squamous intraepithelial lesions. Since the overexpression of p16INK4a protein depends on the presence of transcriptionally-active HPV, and due to its availability and simple interpretation, it may be considered as a proper marker to diagnose cervical cancer.

**Methods:**

An immunohistochemical analysis of p16INK4a was performed in 72 cervical tissue specimens at Imam Khomeini Complex Hospital (Tehran, Iran) from 2016 to 2018. The performance parameters were calculated and compared using receiving operating characteristics curve (ROC) details.

**Results:**

p16INK4a is significantly up-regulated in the cervical cancer samples in comparison with that in normal samples. Moreover, the ROC data showed the potential ability of p16INK4a under determined conditions as a diagnostic marker for CIN 2–3 staging and invasive cervical cancer. The molecular typing disclosed the attendance of HPV DNA in 44.4% of cases (32/72) with a predominance of HPV type 16.

**Conclusion:**

The molecular biomarker p16INK4a can be a good candidate for the early diagnosis and prognosis of cervical cancer in HPV-infected patients. Considering the increase in the expression level of p16INK4a in cancer and precancer tissues, p16INK4a may be used for early detection of cervical cancer.

## Introduction

Human papillomavirus (HPV) has been known by epidemiological and clinical studies as the main pathogen leading to cervical cancer (1). HPV is a non-enveloped, circular double-stranded DNA virus comprising nearly 8,000 base pairs. To date, about 200 subtypes of HPV have been identified based on their L1 capsid protein, sub-categorized into cutaneous or mucosal subtypes (2, 3). Another classification into low-risk (LR) and high-risk (HR) types can be performed based on the capability of developing malignancy or cancerous. Since now, 20 HPV genotypes have been identified as high risk which causes uterine cervix, anus, vagina, vulva, penis, and head and neck cancers (4). HR-HPV sub-types, particularly oncogenic types 16 and 18 develop cervical precancerous lesions (5). One of the cost-effective tests to diagnose HR-HPV is following up on the expression of p16INKa due to its overexpression in the cervical cancerous tissue. Thus, p16INK4a overexpression may be considered as a surrogate biomarker for the presence of high-risk HPV in cervical cancer. Moreover, the correlation between HPV-16, overexpression of p16INK4A, and pRb negativity in oropharyngeal carcinoma have also been reported (6).

HPV oncoprotein E7 comprises a binding site for retinoblastoma (pRb) that causes inactivation of pRb function. The overexpression of p16INK4a is also occurred in E7 expressing cells, which is probably due to the induction of histone demethylases by HPV E7 (7). Although p16INK4a expresses in individual epithelial cells of the lower genital tract (8), the expression level is higher in cells of high-grade precancerous and cancerous cervical lesions (9, 10).

P16INK4a could be considered as the diagnostic tool when the malignant transformation associated with p16INK4a loss in malignant lesions and it also could be a prognostic tool when the malignant transformation accompanies the p16INK4a overexpression as a result of the pRb failure. Therefore, the survey of the p16INK4a expression in human tumors can be of importance to utilize the p16INK4a immunohisto-chemistry as a diagnostic or prognostic tool. Moreover, there are rare details regarding the subcellular location of p16INK4a, which can help the assessment of p16INK4a overexpression in tumors. Eventually, the information about the p16INK4a expression is required to develop new anticancer drugs which act to restore the p16INK4a functionality as one of the major tumor suppressor (11).

Since incorporating the p16INK4A immunohistochemistry and histopathologic diagnosis examination improves diagnosis of the cervical intraepithelial neoplasia (CIN), p16INK4A immunohistochemistry was assessed as the gold standard for defining the efficiency of cervical cancer screening methods (12).

In the present study, the expression of p16INK4a in different samples for the diagnosis of the precancer and invasive cervical cancer in tissue samples was determined. The receiver operating characteristic (ROC) curve analysis was used finding the discriminative value for discriminating the cervical cancer tissues from the pre-cancer and normal tissues.

## Materials and Methods

### Sample selection and histological analysis

Seventy-two fresh uterine cervix biopsies were fixed in neutral buffered. The whole cervical cancer, precancer, and normal tissue samples were obtained from the cervical tissues of patients with informed consent before operations at Imam Khomeini Complex Hospital (Tehran, Iran) from 2016 to 2018.

Patients were also excluded if they had received any neoadjuvant chemotherapy or intraoperative radiation therapy. Slides were reviewed by a single pathologist in a blinded fashion to provide a “study diagnosis” utilized to determine the performance of the different screening tests. All biopsies diagnosed as normal, precancer (CIN1, CIN2, CIN3), or invasive cancer according to international criteria (13). Then, they were reviewed by a second pathologist, and if the second review as opposed to the first, a third pathologist reviewed the case. Considering 2 out of 3 in agreement, a “consensus diagnosis” was obtained.

This study was approved by the Ethics Committee of Tehran University of Medical Sciences (IR.TUMS.SPH.REC.1395.838).

### Immunohistochemistry

Paraffin blocks from 72 biopsies were selected, so that had sufficient diagnostic material remaining for immunohistochemistry. These specimens were included 36 normal, 18 cervical cancer and 18 precancer samples. Five-micron sections were cut and put onto silane-coated slides (Sigma, St. Louis, MO, USA) and processed for immunohistochemistry (14). Anti-human p16INK4A monoclonal antibody (clone E6H4, Dako, Glostrup, Denmark) was used at a 1:50 dilution. Before incubation with the primary antibody, rehydrated sections were microwaved for 15 min in 0.01 citric acid (pH 6.0) and then washed twice with distilled water (15). Endogenous peroxidase activity was terminated by incubation in methanol containing 0.3% hydrogen peroxide for 20 min. Sections were preincubated with 3% normal horse serum in phosphate-buffered saline for 1 h at room temperature (RT), incubated with primary antibody at 4 °C overnight, followed by a 1 h incubation at RT. The avidin-biotinylatedperoxidase complex detection system was used for immunocytochemical localization (Vectastain ABC kit, Vector Laboratory, Burlingame, CA). Immunostaining was imaged using Liquid DAB Pack (BioGenex, CA). For negative controls, slides were incubated with normal rabbit IgG or preimmune serum instead of primary antibody. P16INK4A staining was categorized as either diffuse comprising all layers of the epithelium or basal comprising only the basal and parabasal cell layers and negative. Both diffuse and basal staining could be strong, moderate, or weak.

### Immunohistochemical evaluation

The microscopic analysis of the slides was separately carried out by two researchers. Digital photographs were recorded with a Nikon Coolpix camera DP12. Quantitative outcomes were stated as the percentage of positive cells per field on total cell count. Only cells within the cervical epithelium were enumerated. The whole section slides were evaluated at 400X magnification and separately assessed by two observers. At least, 200 nuclei were evaluated in each case. The counts were accomplished manually and the percentage of positively stained cells in representative microscopic fields was recorded. The reaction was considered positive for p16INK4a when a dark brown color was seen in the nuclei and/or cytoplasmic compartments.

### Evaluation of immunostaining results

For the quantitative evaluation of p16INK4a staining, the percentage of positive cells was measured and then classified according to nuclear and cytoplasmic staining. Immunoreactivity to p16INK4a was classified into three groups according to the percentage of stained cells; weak, variable and strong corresponding to less than 5% of the cells, 5%–50% of the cells (containing weak and strong areas of intensity), and more than 50% of the cells stained for p16INK4a, respectively.

**Fig. 1:**
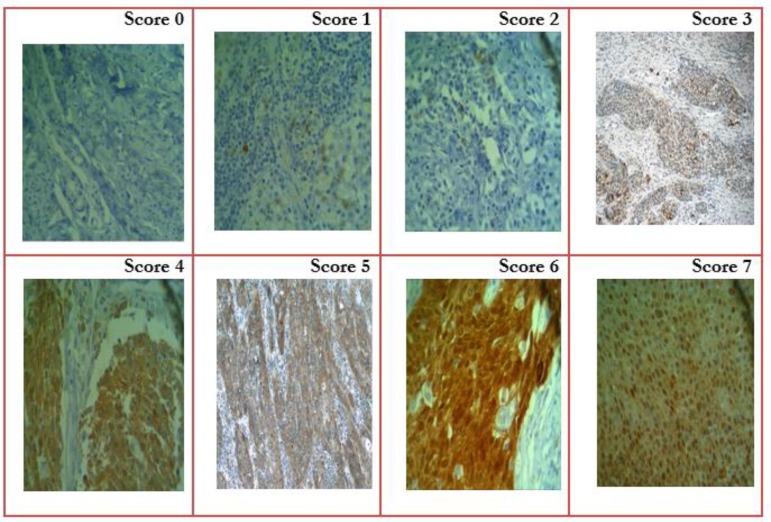
Determination of the p16INK4A semi-quantitative expression according to the criteria described by Allred, based on the Proportion score and Intensity score

Allred score was calculated by measuring the percent of stained cells scored as 0 to 8 and intensity score (weak, intermediate, and strong) (Table 1). The possible values of Allred score are 0 – Allred 0; 1 – Allred 2, 3, 4; 2 – Allred 5, 6; 3 – Allred 7, 8 (Allred score 1 is not possible) (Fig.1).

**Table 1:** The Allred score

***Proportion score (PS)***		***Intensity score (IS)***	
Value	Significance	Value	Significance
0	None	0	None
1	<1%	1	Weak
2	1–10%	2	Intermediate
3	10–33%	3	Strong
4	33–66%		
5	>66%		

### Nested Polymerase Chain Reaction for HPV detection

DNA from each of the selected specimens was extracted with the High Pure DNA extraction kit (Roche, Germany), according to the manufacturer’s protocol. The concentration of DNA was then quantified by NanoDrop ND-1000 spectrophotometer (Thermo Scientific). The quality of the extracted DNA was further checked by PCR amplification of a fragment of the β-globin gene amplified by PC03/PC04 primers (16). The detection of HPV DNA was conducted by two sets of consensus primers, MY09/MY11 and GP5+/GP6+ (16), which amplify a 450 bp and an internal 150 bp region, respectively, in the highly conserved L1 HPV gene. Afterward, the reaction products were electrophoresed on 2% agarose and visualized by SYBR Safe dye.

### Statistical analysis

The Mann-Witney non-parametric test and the one-way ANOVA were carried out to analyze the statistical difference among groups using Graph-Pad Prism (7.0.1) software. A *P*-value of less than 0.05 was considered remarkable. The receiver operating characteristic (ROC) curves were drawn to find the highest sensibility and specificity point. The area under receiver operating characteristic (ROC) curves were calculated using R software (ver. 3.4.4).

## Results

### Patient and control data

The mean age of cervical cancer, precancer, and normal groups were 61 (range: 45–81), 47 (range: 27–57), and 36 (range: 23–49), respectively.

### The p16INK4A expression profile in the tissue samples

**Fig. 2:**
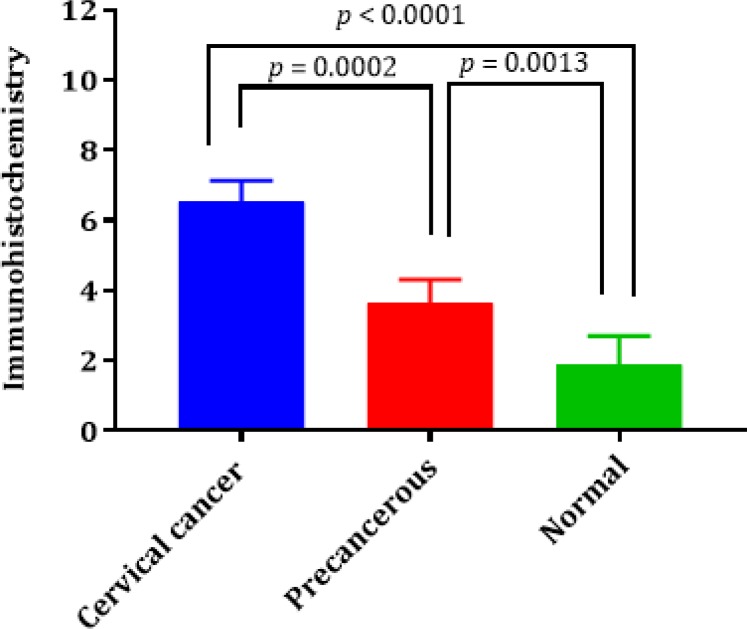
The relative expression level of p16INK4A

The results showed a higher significant expression of p16INK4A in the tissue of cancerous samples than those in normal samples with a *P-*value <0.0001. Moreover, the expression of p16INK4A was remarkably increased in the cancer group in comparison with the precancer group with a *P*-value of 0.0002. The same result was obtained from the comparison between the precancer and normal groups with a p-value of 0.0013 (Fig. 2).

### Receiver operating characteristic (ROC) curve analysis

The ROC curves were generated and the area under curves (AUC) was analyzed to evaluate the diagnostic value of the p16INK4A expression level in cervical cancer, precancer and normal samples (Table 2).

**Table 2:** ROC curve analysis. Area under the curve (AUC) value of p16INK4A in tissue samples

	**Cervical cancer and Normal groups**	**Cervical cancer and Pre-cancer cervical groups**	**Precancer cervical and Normal groups**
p16INK4A	AUC	AUC	AUC
	95% CI : 1	95% CI : 1	95% CI : 0.95
	(1–1)	(1–1)	0.89–1

**Fig. 3:**
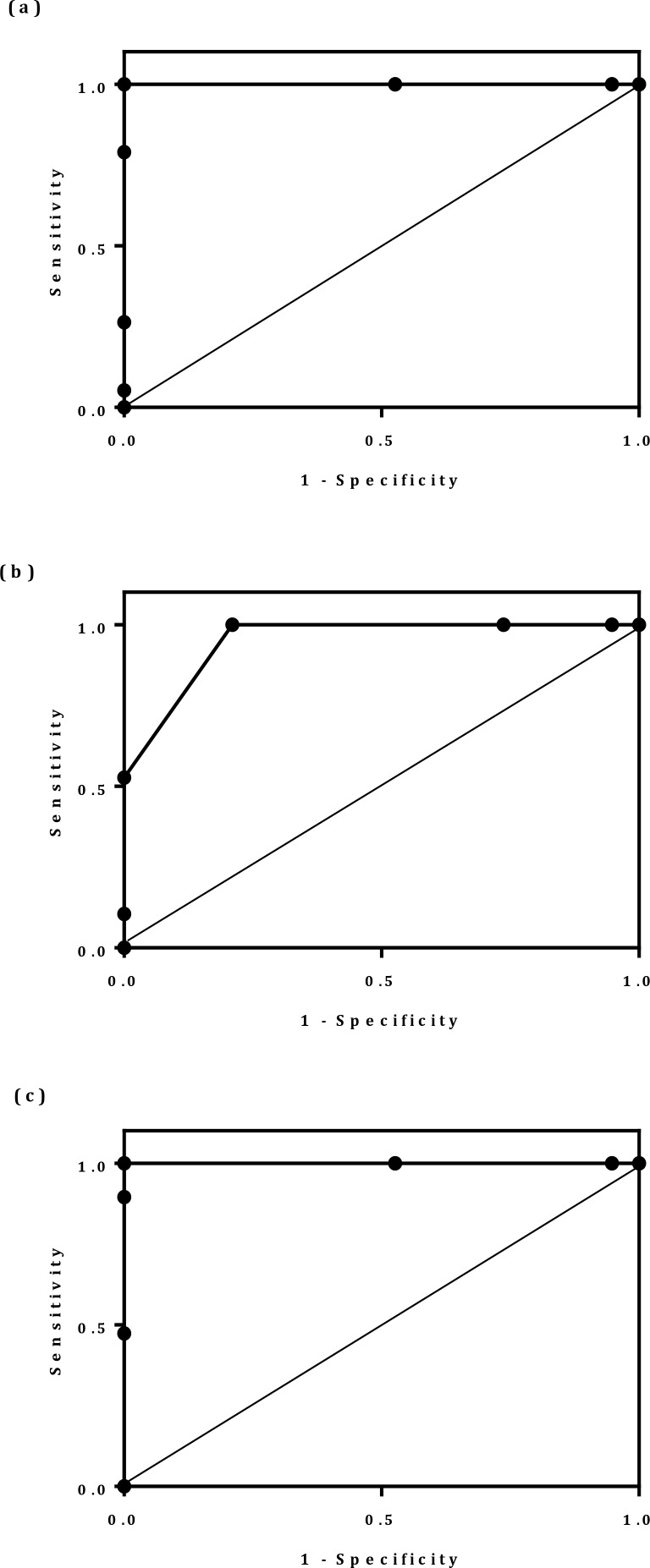
Receiver-operating characteristics (ROC) curve analysis using p16INK4A for discerning different groups in tissue samples. Cervical cancer and normal groups (a), Precancer samples and normal groups (b), cervical cancer and precancer groups (c)

The ROC curves showed that the AUC values in cervical cancer and normal groups were 1(95% CI: 1–1), in cervical cancer and precancer groups were 1 (95% CI: 1–1), and in the precancer and normal groups were 0.95 (95% CI: 0.89–1) (Fig. 3). Therefore, the highest AUC value was obtained from comparing cervical cancer and the normal groups and also cervical cancer and pre-cancer groups. P16INK4A has a strong potential diagnosis value for diagnosing cervical cancer from precancer and normal groups (Table 3).

**Table 3:** The sensitivity and specificity estimation of p16INK4A according to the ROC curves results in tissue samples

***Variable***	***Sensitivity and Specificity***	***Cervical cancer and Normal groups***	***Cervical cancer and Precancer cervical groups***	***Precancer cervical and Normal groups***
p16INK4A	Sensitivity	100	100	100
	Specificity	100	89.47	78.95

### HPV typing

The molecular typing revealed the presence of HPV DNA in 44.4% of the cases (32/72), with a predominance of HPV type 16 (Table 4). Stratification of the pathological status showed that HPV 16 was found in all samples (100%) in the cancer group; HPV 16 and 53 were detected at 50% and 5.5%, respectively, in the precancer group; and HPV 16, 66 and 68 were identified at 5.5%, 2.7%, and 2.7%, respectively, in the normal group.

**Table 4:** The distributions of the HPV genotypes

***Groups***	***Virus genotype***	***Percentage***
Cancer (18)	HPV-16	18(100)
Precancer (18)	HPV-16	9 (50)
	HPV-53	1 (5)
Normal (36)	HPV-16	2 (5.5)
	HPV-66	1 (2.7)
	HPV-68	1 (2.7)

## Discussion

In the present study, the overexpression of p16INK4a in the cervical cancer samples determining by the percentage of positive squamous cells per category was reported. Moreover, to test the predictive power of p16INK4a as a diagnostic marker for precancer and invasive cervical cancer, ROC curves were applied. ROC analysis and calculation of AUC specified the variance of sensitivity and specificity (17). A cut-off value of 54.43% was established and relatively high sensitivity (100%), specificity (100%) were obtained, Accordingly, p16INK4a could be an applicable surrogate marker to discern CIN from other similar tumors and assess the risk of CIN 2–3. These findings are agreement with previous reports regarding the marker potential of p16INK4a for prediction of CINs (18–21). A dramatic increase in the p16Ink4a expression has been reported in the transformation from normal tissue to preneoplastic lesions, and also from preneoplastic lesions to carcinoma in several types of cancer (22–26). The p16INK4A overexpression has been reported at the invasive front of endometrial, colorectal and basal cell carcinoma (27–30).

The p16INK4A upregulation was associated with the expression of other molecules such as the γ2 chain of laminin 5 and β-catenin related to invasive status (27, 28, 31). Furthermore, in vitro studies have demonstrated that p16INK4A is involved in the regulation of matrix-dependent cell migration (32), in glioma invasion (33). Moreover, p16INK4a expression has been reported in many cases of endometrial adenocarcinomas (34–37). The concurrent evaluation of HPV status and p16INK4a expression in en-dometrial carcinomas have been evaluated in only a few cases (34, 35, 37). P16INK4a is known as a common immunohistochemical marker in gynecologic pathology. The nuclear and diffuse cytoplasmic expression of p16INK4a in squamous cell carcinomas of the female genital tract are extremely accompanied by high-risk HPV infection and neoplasms of cervical origin (38). Similar nuclear staining for p16INK4a leads to a change in the cytoplasmic intensity corresponding to the CIN grade. This finding suggests that the hyper-synthesis of p16INK4a in higher grade lesions is a reflection of the overexpression of p16INK4a in the cytoplasm (21). Consistent with other previous studies, a continuous staining pattern from the basement membrane was found that expanded upward in proportion to the lesion grade (39). The staining pattern may be considered as a reliable variable to interpret the p16INK4a rather than other signal intensity determination. Moreover, p16INK4a expression was introduced as an applicable marker to the diagnosis of CIN2 (40). However, these reports only showed the sensitivity and specificity of p16NK4a. In order to establish the best cut-off point for p16NK4a, the ROC curves were established. On the other hand, p16INK4a upregulates as a result of HRHPV E7 expression in proliferating cells (7, 41–47). The increase in intracellular expression of p16INK4A is observed upon the binding of HRHPV derived E7 oncoproteins to the retinoblastoma gene product. Considering the association between HR-HPV and cervical cancers, the over-expression of p16INK4A in HPV-induced neoplasia is expected (19, 48–52). Therefore, based on the immunohistochemical analysis in neoplastic cervical lesions, diffuse p16INK4a positivity can be considered as an indicative marker for the presence of HR-HPVs (50).

## Conclusion

The expression level of p16INK4A changes in cervical cancer cells. The identification of p16INK4A is beneficial to cancer prognosis and early treatment. The up-regulation of p16INK4A in the tissue is a noteworthy biomarker for the diagnosis of HPV-associated cervical cancer. Molecular typing manifested the dominant presence of HPV 16 DNA in cervical cancerous cells. Therefore, our results can help to identify the possible biomarker for HPV-induced cancers.

## Ethical considerations

Ethical issues (Including plagiarism, informed consent, misconduct, data fabrication and/or falsification, double publication and/or submission, redundancy, etc.) have been completely observed by the authors.
